# Antioxidative, Antifungal and Additive Activity of the Antimicrobial Peptides Leg1 and Leg2 from Chickpea

**DOI:** 10.3390/foods10030585

**Published:** 2021-03-11

**Authors:** Marie-Louise Heymich, Laura Nißl, Dominik Hahn, Matthias Noll, Monika Pischetsrieder

**Affiliations:** 1 Food Chemistry, Department of Chemistry and Pharmacy, Friedrich-Alexander Universität Erlangen-Nürnberg (FAU), Nikolaus-Fiebiger-Str. 10, 91058 Erlangen, Germany; marie-louise.heymich@fau.de (M.-L.H.); dominik.hahn@fau.de (D.H.); 2 Institute for Bioanalysis, Department of Applied Sciences, Coburg University of Applied Sciences and Arts, Friedrich-Streib-Str. 2, 96450 Coburg, Germany; lauranissl@web.de (L.N.); matthias.noll@hs-coburg.de (M.N.)

**Keywords:** antifungal activity, antimicrobial peptides, antioxidative peptides, chickpea peptides, cytotoxicity, Leg1, Leg2

## Abstract

The fight against food waste benefits from novel agents inhibiting spoilage. The present study investigated the preservative potential of the antimicrobial peptides Leg1 (RIKTVTSFDLPALRFLKL) and Leg2 (RIKTVTSFDLPALRWLKL) recently identified in chickpea legumin hydrolysates. Checkerboard assays revealed strong additive antimicrobial effects of Leg1/Leg2 with sodium benzoate against *Escherichia coli* and *Bacillus subtilis* with fractional inhibitory concentrations of 0.625 and 0.75. Additionally, Leg1/Leg2 displayed antifungal activity with minimum inhibitory concentrations of 500/250 µM against *Saccharomyces cerevisiae* and 250/125 µM against *Zygosaccharomyces bailii*. In contrast, no cytotoxic effects were observed against human Caco-2 cells at concentrations below 2000 µM (Leg1) and 1000 µM (Leg2). Particularly Leg2 showed antioxidative activity by radical scavenging and reducing mechanisms (maximally 91.5/86.3% compared to 91.2/94.7% for the control ascorbic acid). The present results demonstrate that Leg1/Leg2 have the potential to be applied as preservatives protecting food and other products against bacterial, fungal and oxidative spoilage.

## 1. Introduction

Peptides occur naturally in many foods or can be formed after the enzymatic or microbiological hydrolysis of food proteins. Dietary peptide fractions feature a huge structural variety and diverse bioactive properties including, among others, antioxidative, antihypertensive, antimicrobial, anti-inflammatory, anticancer and antithrombotic activities [1]. Particularly antimicrobial peptides (AMPs) from food sources could be a promising alternative to conventional preservatives. It has been postulated that AMPs interact specifically with the bacterial cell membrane and lead to its disintegration, for example by the formation of pores [2]. Thus, AMPs are active against a wide range of bacteria and are less likely to evoke antimicrobial resistance [2,3]. The risk of toxic side effects is low so that food-derived AMPs are generally considered as safe. Similarly, enzymatically generated AMPs from food proteins are equivalent to products of gastrointestinal digestion and most probably safe. So far, however, only one peptide is utilized as food preservative, namely nisin, which is of bacterial origin [4].

**Figure 1:**
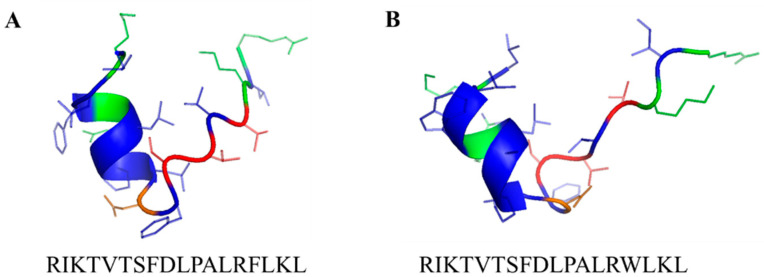
Peptide sequence and secondary structure of (**A**) Leg1 and (**B**) Leg2 [5] predicted with *I‑Tasser* (https://zhanglab.ccmb.med.umich.edu/I-TASSER/, accessed on 22 May 2019) and generated by *PyMOL* (http://www.pymol.org/, accessed on 13 January 2021). Hydrophobic amino acids are colored in blue, polar amino acids in red, positively charged amino acids in green and negatively charged amino acids in orange.

Recently, the antimicrobial peptides Leg1 (RIKTVTSFDLPALRFLKL) and Leg2 (RIKTVTSFDLPALRWLKL) (Figure 1) were identified in enzymatic hydrolysates of chickpea storage protein [5]. Both peptides were active against 16 strains of food spoilage bacteria and food pathogens. With minimum inhibitory concentrations (MIC) down to 15.6 µM, the peptides proved 10–1000-fold more active than conventional preservatives under the applied conditions.

Besides preventing bacterial spoilage, however, bioactive peptides could improve the food’s shelf life by antioxidative activity or cooperative effects with other preservatives. Additionally, peptides with antifungal activity [6] would be an asset against waste. Fungal spoilage of foods and beverages with high sugar contents, low pH and low water activities leads to major economic problems [7]. Globally, only a few yeast species are responsible for major losses in processed foods [8]. Among others, yeasts of the genera *Zygosaccharomyces* and *Saccharomyces* spoil food and drinks on a large scale. Especially *Zygosaccharomyces (Z.) bailii* pose a problem, because they show extreme osmotolerance, the ability to ferment hexose sugars and resistance to weak-acid preservatives [9,10,11].

Mold and yeast species differ in cell wall and membrane organization among each other and from bacteria, so that antimicrobial and antifungal activities of peptides do not necessarily coincide. The aim of the present study was to investigate if the newly detected food-derived AMPs Leg1 and Leg2 from chickpea [5] have an impact on other food spoilage processes including oxidation and fungal growth. Considering the different mechanisms of action of AMPs compared to low-molecular preservatives, we examined also if Leg1 and Leg2 may potentiate the activity of the conventional preservative sodium benzoate. Cooperative effects between combined preservatives may reduce the single concentrations required for effective food preservation.

## 2. Materials and Methods

### 2.1. Peptide Synthesis

Leg1 (RIKTVTSFDLPALRFLKL, molecular weight (MW) 2118.59 g/mol) and Leg2 (RIKTVTSFDLPALRWLKL, MW 2157.62 g/mol) were commercially produced by solid-phase synthesis at ChinaPeptides Co. (Shanghai, China) and provided as trifluoroacetic acid salt-lyophilized powder (purity > 95%). The quality of the products was verified as described before by ultrahigh-performance liquid chromatography (UHPLC; Ultimate 3000 RS, Thermo Fisher Scientific, Idstein, Germany) coupled to a TripleQuad 6500^+^ mass spectrometer (AB Sciex, Darmstadt, Germany), equipped with duospray ion source [5]. The correct purity and mass were confirmed by enhanced mass spectrum full-scan analysis and the correct peptide sequence by enhanced product ion scan. The peptides were solved in sterile water (2 mM) and stored at −20 °C until use.

### 2.2. Bacterial and Fungal Strains and Culture Conditions

*Escherichia (E.) coli* NEB 5α were obtained from New England Biolabs (Ipswich, MA, USA) and *Bacillus (B). subtilis* ATCC 6051 from the Leibniz Institute DSZM-German Collection of Microorganisms and Cell Cultures (Braunschweig, Germany). The bacteria were grown in nutrient broth (8 g/L) at 37 °C for 24 h under shaking in a temperature-controlled incubator shaker (Innova42R, New Brunswick, New York, NY, USA) as described by Heymich et al. [5]. DSZM also supplied the mold strain *Aspergillus (A.) niger* DSM 12634 and the yeasts *Pichia (P.) membranifaciens* DSM 70633, *Saccharomyces (S.) cerevisiae* DSM 70499 and *Z. bailii* DSM 70492. *A. niger* was transferred to potato dextrose bouillon (PDB) agar plates (26.5 g/L PDB). The other fungal strains were cultured on universal medium for yeasts agar plates (1.5% (*w*/*v*) yeast/molds (YM) agar; 3 g/L yeast extract, 3 g/L malt extract, 5 g/L peptone from soybeans, 10 g/L glucose). All media components were obtained from Carl Roth (Karlsruhe, Germany).

The concentration of the bacterial suspension was calculated by optical density at 600 nm (OD_600_) using a UV-visible spectrophotometer (Genesys 10S, Thermo Fisher Scientific) with nutrient broth as reference. The colony-forming unit rates per milliliter (cfu/mL) were calculated for *E. coli* (1.0 OD = 8 × 10^8^ cfu/mL) and *B. subtilis* (1.0 OD = 8 × 10^9^ cfu/mL) and the suspensions were diluted with nutrient broth to the appropriate concentration for each assay. Yeast inoculum suspensions were prepared in YM medium as described by Arendrup et al. [12] and were adjusted to a count of 9.0 × 10^4^ to 1.0 × 10^5^ cfu/mL (corresponding to OD_620_ of 0.12 to 0.15 measured by a UV-visible spectrophotometer, Analytik Jena, Jena, Germany). The mold inoculum was prepared in PDB and adjusted to a cell number of 1.0 × 10^6^ spores/mL by a Neubauer chamber (depth 0.1 mm; Paul Marienfeld, Lauda-Königshofen, Germany) as described by Arendrup et al. [13].

### 2.3. Checkerboard Assay to Test for Combinatorial Effects of Leg1/Leg2 and Sodium Benzoate

To test for synergistic or additive effects, a checkerboard assay with resazurin was performed according to Sarker et al. [14] with small modifications. A resazurin solution was prepared solving one tablet (Thermo Fisher Scientific) in 50 mL of water, followed by sterile filtration (pore size 0.22 µm). The peptides Leg1, Leg2 or nisin (2.5%, Sigma-Aldrich, Steinheim Germany) (0–500 µM each) and sodium benzoate (0–160,000 µM, Sigma-Aldrich) were tested against *E. coli* and *B. subtilis* separately or in combination. Twenty-five µM ampicillin (ampicillin sodium salt, Carl Roth) was used as positive and sterilized water as negative control. Either a combination of 25 µL of peptide solution and 25 µL of sodium benzoate or 50 µL of each single substance was transferred to 96-well microtiter plates (Greiner BioOne, Kremsmünster, Austria) in a two-fold dilution. Then 30 µL of nutrient broth, 10 µL of resazurin solution (6 mg/mL) and 10 µL of the bacterial suspension (1–5 × 10^6^ cfu/mL) were added. After an incubation time of 16 h at 37 °C, the plates were measured at OD_570_ using a microplate reader (µQuant, Biotek, Bad Friedrichshall, Germany). MIC was defined as the lowest concentration at which no bacterial growth as indicated by a color change from blue to pink was observed. The fractional inhibitory concentration (FIC) index was calculated by the sum of the divided MIC values as follows:

(1) $$\mathrm{FIC}={\mathrm{FIC}}_{A}+{\mathrm{FIC}}_{B}=\frac{{\mathrm{MIC}}_{\mathrm{AB}}}{{\mathrm{MIC}}_{A}}+\frac{{\mathrm{MIC}}_{\mathrm{BA}}}{{\mathrm{MIC}}_{B}}.$$

where MIC_A_ is the MIC of the single peptides Leg1, Leg2, or nisin, respectively, MIC_B_ is the MIC of sodium benzoate alone, and MIC_AB_ and MIC_BA_ are the MIC concentrations of the substances in combination. FIC values <0.5 were interpreted as synergy, 0.5 < FIC > 2.0 as additivity, 2.0 < FIC > 4.0 as indifference and FIC >4.0 as antagonism. All experiments were performed in triplicates and repeated twice.

### 2.4. Antifungal Susceptibility Tests

The antifungal assays were performed as described previously by Arendrup et al. [12,13] with minor modifications. The peptides Leg1, Leg2, and nisin were tested in a concentration range of 1–1000 µM and sodium benzoate at 156–160,000 µM. The MIC values of *A. niger*, *P. membranifaciens*, *Z. bailii*, and *S. cerevisiae* were determined in vitro in triplicates in a 96-well microtiter plate setup by a broth microdilution method. Each well contained 50 µL of peptides/sodium benzoate, 25 µL of fourfold concentrated medium (YM bouillon or PDB) and 25 µL of yeast or mold inoculum. The microtiter plates for the yeast susceptibility tests were incubated at 25 °C for 48 h. Because the mold strain *A. niger* grows slowly, it was incubated at 25 °C for 72 h. The microtiter plates were analyzed by OD_620_ measurement (FLUOstar Omega, BMG Labtech, Ortenberg, Germany). Growth was defined by ΔOD_620_ ≥ 0.2 compared to the negative control and the MIC was the lowest concentration resulting in ΔOD_620_ < 0.2.

### 2.5. Flow Cytometry Measurement of Antifungal Activity

The antifungal susceptibility tests of *S. cerevisiae* and *Z. bailii* were complemented with flow cytometry measurements using a NovoCyte Flow Cytometer (Acea Biosciences, San Diego, CA, USA). Live/dead staining was performed with the fluorescent dye SYBR Green I (10,000× concentrate in dimethyl sulfoxide (DMSO); Lonza Group, Basel, Switzerland) and propidium iodide (PI; 1 mg/mL; Biotium, Fremont, CA, USA). To reduce background noise, the threshold value of the forward scatter was set to a lower limit of 10,000 (channel value). A photomultiplier with a band pass filter of 530/30 nm was used to collect the green fluorescence of SYBR, while the red fluorescence of PI was detected using a band pass filter of 660/20 nm. For each sample, 20 µL was collected at a flow rate of 3000 events per second. Analysis and gating of data were performed using the Novo Express software 1.2 (Acea Biosciences). Because a combination of forward scatter and sideward scatter was used to discriminate cells from the background, a species-specific gate for cell detection was defined. For the evaluation of viability, the gates were set manually and verified by running unstained, single-stained and dual-stained samples or negative controls containing no cells. Quadrants were associated with cell membrane integrity (intact: SYBR Green I positive but PI negative; not intact: both SYBR Green I and PI positive). Cell concentrations with and without intact cell membrane integrity were calculated from each sample volume as events in the respective gates. Flow cytometry measurements were performed in triplicates.

### 2.6. Assays for Antioxidative Activity

The antioxidative activity was tested in 96-well microtiter plates using the 2,2-diphenyl-1-picrylhydrazyl (DPPH) assay according to Chen et al. [15] and the reducing power assay according to Chu et al. [16] with small modifications. After mixing 50 µL of peptide solution with 50 µL of DPPH (2 mM solved in ethanol, Sigma-Aldrich), the sample was incubated overnight under light exclusion. Afterwards a microplate reader measured the absorption (A) at 515 nm monitoring the discoloration of purple to light yellow. A mixture of water and ethanol without DPPH was used as blank. The antioxidative activity in percent (scavenging activity) was calculated with the following equation:

(2) $$\mathrm{Scavenging}\;\mathrm{activity}\;\left(\%\right)=100-\frac{\left(A_{\mathrm{Sample}}-A_{\mathrm{Blank}}\right)\times 100}{A_{\mathrm{neg}.\mathrm{Control}}}.$$

To quantify the reducing power, 50 µL of the peptide solution was mixed with 50 µL of phosphate buffer (0.2 M, pH 6.6) and 50 µL of 1% potassium ferricyanide solution (Merck, Darmstadt, Germany). The mixture was incubated for 20 min at 50 °C, followed by the addition of 50 µL of trichloroacetic acid (10%, BioXtra, Sigma-Aldrich) and a centrifugation step (857× *g*, 10 min). The 96-well microtiter plate was prepared with 50 µL each of sample mixture, water and 0.1% anhydrous ferric chloride solution (98%, Sigma-Aldrich), incubated for 5 h in the dark and measured at 700 nm using a microplate reader. The reducing power (in percent) and, therefore, the formation of the Prussian blue complex was calculated as follows:

(3) $$\mathrm{Reducing}\;\mathrm{power}\;\left(\%\right)=100-\left(A_{\mathrm{neg}.\mathrm{Control}}/A_{\mathrm{Sample}}\right)\times 100$$

In both assays, water was used as negative and ascorbic acid as positive control. Peptides and positive control were tested in twofold dilution from 15.6 to 1000 µM. Additionally, the free amino acids tryptophan, tyrosine, histidine, cysteine, proline, and phenylalanine were tested using the same concentration range. The antioxidative activity was determined in triplicates and the experiments were repeated twice.

### 2.7. Cell Culture

Human colorectal adenocarcinoma cells (Caco-2) were cultured in GlutaMax minimum essential medium maintained in 75 cm^2^ sterile flasks (Nunc EasyFlask, Thermo Fisher Scientific) at 37 °C in a saturated humidity atmosphere containing 5% CO_2_ and 95% air. The medium was supplemented with 57 mL of fetal bovine serum (not inactivated, 10%), 5.8 mL of sodium pyruvate (100 mM), 5.8 mL of nonessential amino acids (100×) and 5.8 mL of penicillin (10,000 IU)/streptomycin (10,000 μg/mL)/amphotericin B (25 μg/mL). The medium and all supplements for cell culture were obtained from Life Technologies (Darmstadt, Germany).

### 2.8. MTT Cytotoxicity Assay

The cytotoxicity of the test substances was assessed in the 3-(4,5-dimethylthiazol-2-yl)-2,5-diphenyltetrazoliumbromid (MTT) assay [5]. Caco-2 cells were incubated with different concentrations of Leg1 (7.8–2000 µM), Leg2 (7.8–2000 µM), nisin (7.8–2000 µM), sodium benzoate (313–80,000 µM), potassium sorbate (625–160,000 µM, Acros Organics, brand of Thermo Fisher Scientific), and sodium nitrite (156–40,000 µM, Sigma-Aldrich). Water was used as negative and 10% DMSO as positive cytotoxic control. The negative control was set as 100% cell viability and the cytotoxicity of the test substances was calculated referring to the negative control. Cytotoxicity was determined in triplicates.

### 2.9. Statistical Analysis

GraphPad PRISM 8 and OriginPro 2019 (OriginLab Corporation, Northampton, MA, USA) were used for statistical analysis. The results were depicted as the mean of triplicates ± standard deviation. The results were compared to the negative control using one-way analysis of variance (ANOVA) with Tukey’s Honest Significant Different (HSD) test for pairwise comparison (95% confidence interval). The levels of significance were * *p* < 0.05, ** *p* < 0.01, *** *p* < 0.001, **** *p* < 0.0001.

## 3. Results and Discussion

A previous study demonstrated the antibacterial activity of Leg1 and Leg2 against a wide range of food spoilage bacteria and food pathogens [5]. Based on the structure of these peptides and the reported mode of action of the antimicrobial effects, we hypothesized that Leg1 and Leg2 may show additional activity against food spoilage. In particular, we tested for cooperative activity with sodium benzoate and antifungal as well as antioxidative activities.

### 3.1. Cooperative Effects of Leg1 and Leg2 with Sodium Benzoate on Bacterial Growth

**Figure 2:**
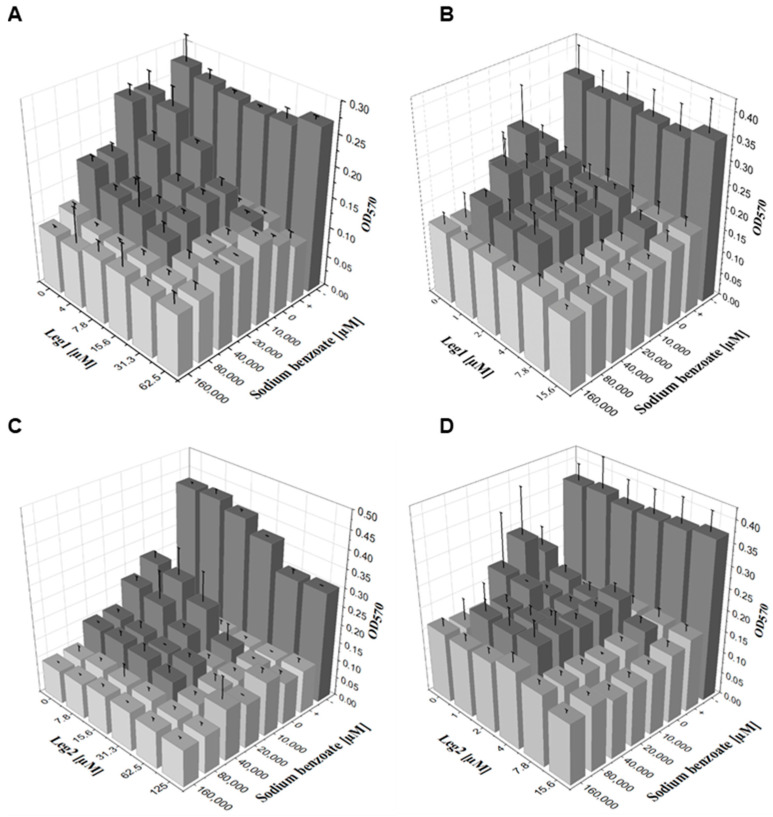
Results of a checkerboard assay using Leg1 or Leg2 combined with sodium benzoate against (**A**,**C**) *E. coli* and (**B**,**D**) *B. subtilis* to determine the fractional inhibitory concentration (FIC) indices. Water was used as negative control (−) and ampicillin (+) as positive control. The optical density is displayed as mean ± SD of triplicates measured at 570 nm. Bacterial growth is indicated in dark grey.

While antimicrobial agents with the same mechanism of action compete and lead to indifferent effects on antimicrobial activity, preservatives with different mechanisms of action may have at least additive or even synergistic impact. Organic acids like sodium benzoate or potassium sorbate are bacteriostatic agents. After passing the cell membrane in undissociated form, they dissociate inside the cell and inhibit bacterial growth by pH-induced stress [17]. In contrast, AMPs act as membrane-disrupting bactericidal agents that interact with the bacterial membrane and form pores eventually killing the bacteria [2]. These pores may facilitate the entry of sodium benzoate into the cell and consequently lead to cooperative antimicrobial activity. To test for possible cooperative effects, we added the peptides Leg1 or Leg2 to the preservative sodium benzoate and determined the antimicrobial effects of all pairings against *E. coli* and *B. subtilis* in a checkerboard assay measuring the OD_570_ (Figure 2). In combination with Leg1 and Leg2, the MIC values of sodium benzoate against *E. coli* and *B. subtilis* were considerably lower indeed. For example, 31.3 µM Leg1/Leg2 reduced the MIC value of sodium benzoate against *E. coli* by a factor of eight.

To quantify combinatorial effects of Leg1 and Leg2 with sodium benzoate, the FIC index was calculated. FIC values of 0.625 for *E. coli* (Table 1) and 0.75 for *B. subtilis* (Table 2) imply strong additive effects in all combinations. The results were very similar to the cooperative activity of nisin, which was tested in the same way as Leg1 and Leg2 (Table 1 and Table 2) indicating a general cooperative mechanism of AMPs and sodium benzoate.

Previous studies demonstrated that other AMPs acted synergistically/additively in combination with conventional antibiotics [18,19]. Additionally, Lòpez-Expòsito et al. showed similar effects for milk peptides in combination with the preservative peptide nisin against Gram-positive bacterial strains (*Listeria monocytogenes* and *Staphylococcus epidermidis*) [20]. Whereas Stanojevic et al. observed indifferent activity against most bacterial strains by the combined preservatives sodium benzoate and potassium sorbate, the combination of these organic acids with sodium nitrite had synergistic effects [21]. The simultaneous application of the antimicrobial peptides Leg1/Leg2 with conventional preservatives, such as sodium benzoate, may allow reducing their dose and may address a wider spectrum of microorganisms.

### 3.2. Antifungal Activity of Leg1 and Leg2

**Figure 3:**
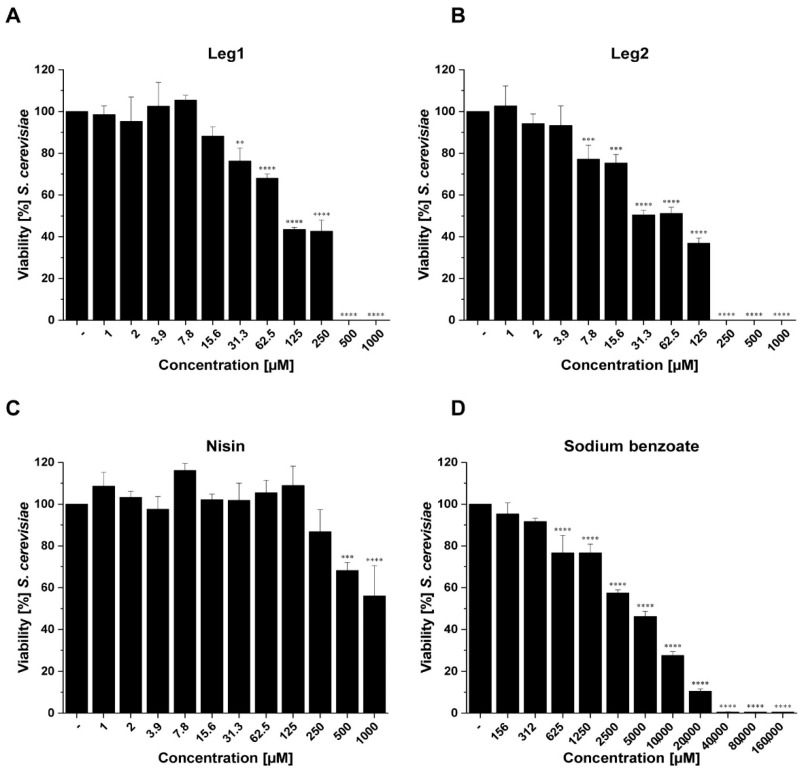
Results of the flow cytometry measurement of antifungal activity using (**A**) Leg1, (**B**) Leg2, (**C**) nisin, and (**D**) sodium benzoate against *S. cerevisiae*. Water (−) was used as negative control. Viability (as mean ± SD of triplicates) is expressed as percentage in relation to the negative control. The levels of significance were ** *p* < 0.01, *** *p* < 0.001, **** *p* < 0.0001.

**Figure 4:**
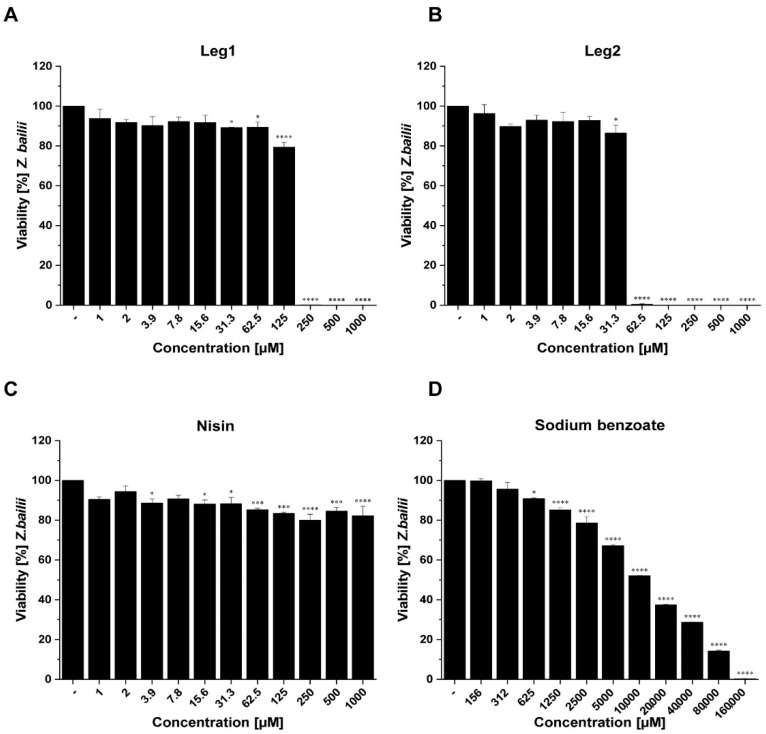
Results of flow cytometry measurements of antifungal activity using (**A**) Leg1, (**B**) Leg2, (**C**) nisin, and (**D**) sodium benzoate against *Z. bailii*. Water (−) was used as negative control. Viability (as mean ± SD of triplicates) is expressed as percentage in relation to the negative control. The levels of significance were * *p* < 0.05, *** *p* < 0.001, **** *p* < 0.0001.

The composition of fungal cell membranes differs fundamentally from bacterial membranes [22]. Therefore, the efficacy of cell-penetrating antimicrobial peptides against bacteria cannot be easily extrapolated to fungi. In order to determine the activity of Leg1 and Leg2 against fungi, a microdilution assay was applied. Both peptides showed fungistatic activity against *S. cerevisiae* and *Z. bailii*, but not against *P. membranifaciens* or *A. niger* (Supplementary Materials, Tables S1 and S2). In addition, flow cytometry analysis was performed to confirm the fungistatic effects of Leg1 and Leg2 against *S. cerevisiae* and *Z. bailii* and to differentiate fungistatic, fungicidal, or fungilytic modes of action of both peptides. Fungistatic agents inhibit the cell division [23], whereas fungicidal agents cause cell death [24], which can be distinguished by fluorometric staining. Flow cytometry determined fungistatic effects of Leg1 and Leg2 against *S. cerevisiae* and *Z. bailii*; Leg2 had lower MIC values (250 µM and 125 µM) than Leg1 (500 µM and 250 µM). Both AMPs also showed fungicidal effects indicated by lower cell numbers than the inoculum concentration. In general, the fungicidal effect was more pronounced against *Z. bailii* than *S. cerevisiae*. Even at concentrations below MIC, Leg1/Leg2 significantly reduced the number of yeast cells compared to the controls (Figure 3 and Figure 4).

Leg1 and Leg2 are probably membrane-active peptides [5] and the variations in antifungal activity might be caused by different architectures of the cell envelope, which is crucial for cell viability, morphology and protection against stressors like AMPs. Fungal cell walls have multifaceted composition and organization and are mainly composed of glucans, chitin, and glycoproteins [25]. The cell walls of the filamentous fungi *Aspergillus* spp. have a higher chitin content than those of *S. cerevisiae* and contain high amounts of melanin, which contribute to fungal virulence [26,27]. Grillitsch et al. observed clear differences in the cell membrane structure, for example, the degree of fatty acid saturation and ergosterol levels of *P. pastoris* compared to *S. cerevisiae* [28], which could be the reason why Leg1 and Leg2 had no antifungal effect against *P. membranifaciens*. In contrast, *S. cerevisiae* and *Z. bailii* both belong to the family *Saccharomycetaceae* and have high phylogenetic correlation and a high percentage of identical gene sequences [29,30]. Additionally, Nguyen et al. reported a similar general composition of the cell wall of *Z. bailii* and *S. cerevisiae* containing mannoprotein (24–35%), chitin (0.6–3%), alkali-soluble (34–37%) and alkali-insoluble glucan (20–37%) [31].

The present results demonstrate antifungal effects of the AMPs Leg1 and Leg2 against members of the family *Saccharomycetaceae*, but not the families *Pichiaceae* or *Trichocomaceae*. For comparison, sodium benzoate, a common weak-acid preservative with fungistatic effects [32] and nisin were included in the present study. Nisin is known for its antimicrobial effect against a number of Gram-positive bacteria and also for inhibiting the outgrowth of spores [33]. In general, nisin has no inhibitory effect on yeasts and filamentous fungi, but Dielbandhoesing et al. showed that *S. cerevisiae* is sensitive against nisin in certain stages of the cell cycle [34]. Nisin significantly reduced the cell numbers of both *S. cerevisiae* and *Z. bailii*, but had no fungicidal effect (Figure 3 and Figure 4). Fungistatic effects on both strains were found after treatment with high concentrations of sodium benzoate (*S. cerevisiae*, 40,000 µM; *Z. bailii*, 160,000 µM). This result is not surprising because *Z. bailii* is known to be very resistant to weak-acid preservatives [10].

In concentrations ≥125 µM (Leg1) and ≥62.5µM (Leg2), blank controls of Leg1 and Leg2 without inoculum showed a concentration-dependent increase of OD_620_ values in the microdilution assay (Supplementary Materials, Figure S1). However, no elevated OD_620_ values were observed in other assays such as the antimicrobial assay, so that matrix-specific peptide aggregation can be assumed, dependent on the pH or matrix components [35,36,37,38]. Further studies are required to determine if the observed antifungal susceptibility is dependent on the peptide aggregation.

### 3.3. Antioxidative Activity of Leg1/Leg2

Besides bacterial and fungal activity, oxidation reactions are a common cause of food spoilage. Many peptides have antioxidative properties, particularly if they contain antioxidant amino acids; the present study examined therefore possible antioxidative effects of Leg1 and Leg2 in radical scavenging- (DPPH) and reducing-power assays. Tryptophan, phenylalanine, and proline are part of the Leg1 and Leg2 sequences and have antioxidative properties. Therefore, the assay covered these single amino acids to evaluate their contribution to the activity of the peptides. Additionally, the antioxidative amino acids histidine, cysteine, and tyrosine [39] were included for comparison and as further positive controls. Table 3 displays the radical scavenging activity of Leg1/Leg2 and the selected single amino acids. High concentrations of Leg2 (500 µM, 1000 µM) showed an antioxidative activity of about 91.5–72.2%. The activity decreased dose-dependently down to 5.3% at a concentration of 15.6 µM. At the higher concentrations, the antioxidative impact of Leg2 was in the same range as the effects of ascorbic acid and the most active amino acids tyrosine and cysteine. In the lower concentration range, Leg2 was less active than ascorbic acid, tyrosine, and cysteine, but still more active than histidine. With a maximum activity of about 29.3%, Leg1 acted as a weak radical scavenger. Aromatic amino acids like phenylalanine and tryptophan act as radical scavenger by donating hydrogens or electrons to electron-deficient radicals. The single amino acid tryptophan had 60.8–17.6% scavenging activity, whereas phenylalanine reached only 22.8%. Because Leg2 differs from Leg1 solely in the exchange of phenylalanine by tryptophan, the increased antioxidative activity of Leg2 can be attributed to tryptophan. However, Leg2 showed a higher activity compared to tryptophan alone. Therefore, the other hydrophobic amino acids, such as proline, alanine, leucine, isoleucine or effects by the neighboring amino acids are likely to play an additional role.

The results of the ferric reducing assay (Table 4) confirmed the strong antioxidative activity of Leg2 observed by the radical scavenging assay. With a maximum activity of 86.3%, the reducing power of Leg2 was only slightly lower than the effect of ascorbic acid (maximum 94.7%) and similar to cysteine, tyrosine and histidine (maxima 90.2%, 87.1% and 85.4%) throughout the whole concentration range.

In contrast to its low radical scavenging activity, Leg1 showed also notable reducing activity with a maximum of about 70.3% indicating that Leg1 may contribute to the antioxidative activity by reducing rather than radical scavenging mechanisms. In the reducing assay, tryptophan was also more active than phenylalanine. This finding is in accordance with the literature [40] and may explain the stronger effects of Leg2 compared to Leg1. The reducing activities of Leg2 and tryptophan (maximum 92.7%) are comparable suggesting that this amino acid is mainly responsible for the effect of Leg2. In contrast, Leg1 shows a higher activity compared to the single amino acid phenylalanine (maximum 22.8%).

The presence of hydrophobic amino acids and especially aromatic amino acids (tryptophan, tyrosine, phenylalanine) can be the main determinant of the antioxidative activity of peptides [39]. Additionally, the indole ring of tryptophan acts as a strong hydroxyl radical scavenger. The higher reducing activity of Leg1 compared to phenylalanine can be explained by cooperative effects of other amino acids, although this phenomenon is not fully understood. For example, Ayala-Niño et al. showed that the presence of tryptophan in peptides is responsible for their radical trapping activity, which can be enhanced by a neighboring arginine [41]. Additionally, the presence of leucine and proline can increase the antioxidative activity by enhancing the hydrophobicity [42]. Thus, the presence of leucine and isoleucine in Leg1and Leg2 may further promote the reducing power of the peptide [43]. Structure-activity relationships were examined in the past addressing, for example, the position of an amino acid in the sequence (c-terminal, n-terminal), the presence of functional groups or the molecular weight [44,45]. However, synergistic effects between amino acids or the influence of the tertiary structure are hardly elucidated, so that the behavior of different sequences in various test assays cannot be predicted yet. Recently, a good correlation between the radical scavenging activity and reducing activity of ovalbumin hydrolysates was reported [46].

Because the antimicrobial peptides Leg1 and especially Leg2 showed antioxidative activity, these peptides could simultaneously protect foods against microbial and oxidative deterioration. Utilizing Leg 1 and Leg 2 as preservatives may thus additionally prevent fat rancidity and color changes. A combined antioxidative and antimicrobial activity was reported for peptide fractions of string beans, whereas the fungus *Candida albicans* was not sensitive [47].

### 3.4. Cytotoxicity against Caco-2 Cells

**Figure 5:**
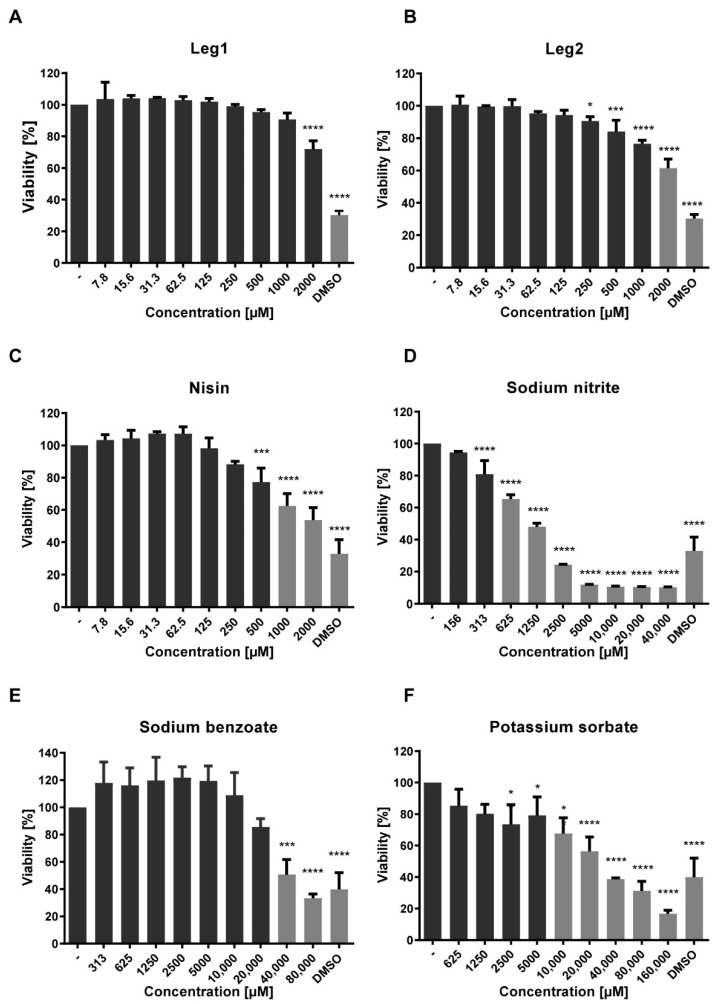
Influence of (**A**) Leg1, (**B**) Leg2, (**C**) nisin, (**D**) sodium nitrite, (**E**) sodium benzoate, and (**F**) potassium sorbate on the viability of Caco-2 cells as determined by the MTT assay. Water (−) was used as negative control and 10% DMSO as positive control. Viability (as mean ± SD of triplicates) is expressed as percentage in relation to the negative control. Cytotoxic effects (viability < 70%) are highlighted in light gray. The levels of significance were * *p* < 0.05, *** *p* < 0.001, **** *p* < 0.0001.

Molecular dynamics simulations attributed the antimicrobial activity of Leg1 and Leg2 to a specific deterioration of the bacterial membrane [5]. The observed antifungal activity may result from a different mechanism, because bacterial and fungal cell membranes differ in structures and compositions. Therefore, an unspecific interaction with different membrane types cannot be excluded, which would imply that Leg1 and Leg2 could have cytotoxic effects against human cells. To assess possible cytotoxic activity, the effect of Leg1 and Leg2 on the viability of human Caco-2 cells was tested in a MTT assay. Local cytotoxic effects of food-derived AMPs in the gastro-intestinal tract are probably more relevant than systemic activity. Therefore, Caco-2-cells were used to determine cytotoxicity, because they originate from the human colon and are often used as a model for the intestinal barrier [48]. For comparison, the approved preservatives sodium benzoate, potassium sorbate, sodium nitrite, and nisin were included in the assay. The test concentration ranges were selected based on the MIC values of the compounds against *E. coli* and *B. subtilis* [5]. Figure 5 displays the results of the MTT assay after an incubation time of 16 h.

According to ISO 10993-5, cytotoxicity is defined by a cell viability <70% compared to the control [49]. Thus, Leg1 was not cytotoxic in any of the applied concentrations, whereas only the highest concentration of Leg 2 (2000 µM) showed a cytotoxic effect with 62% cell viability. In contrast, nisin led to a cell viability <70% and therefore cytotoxic effects at 1000 µM and 2000 µM. Sodium benzoate reduced the cell viability at 40,000 µM, potassium sorbate at 10,000 µM, and sodium nitrite at 625 µM. Thus, the present MTT assay confirmed that Leg1/Leg2 did not interfere with the cell viability of human colon cells in concentrations around the MIC of 62.5 µM against *E. coli* as reported before [5] and of 15.6 µM against *B. subtilis*. Furthermore, no cytotoxic effects were observed at the MICs of potassium sorbate (40,000 µM against *E. coli*, 80,000 µM against *B. subtilis*) and sodium nitrite (20,000 µM against *E. coli*, 40,000 µM against *B. subtilis*), whereas sodium benzoate was cytotoxic at its MIC (40,000 µM against *E. coli*).

So far, the observed MIC values were determined under optimal conditions for bacterial growth [5]. Under actual food storage conditions, when, for example, low temperatures or low oxygen levels pose an additional hurdle for bacterial growth, lower AMP concentrations can be effective. Therefore, the gaps between cytotoxic doses and the applied concentrations in food are probably even larger.

In case of nisin, previous studies determined cytotoxic effects at 298 µM after 4 h against colon cancer cells [50], at 150 µM after 1 h against melanoma cells [51] and at 7.5 µM after 24 h against astrocytoma cells [52]. Vaucher et al. described the cytotoxic activity of the antimicrobial peptide P40, which was comparable to nisin [53]. The low cytotoxicity of Leg1 and Leg2 against human Caco-2 cells indicates a very specific interaction of both AMPs with the bacterial membrane. This observation is in very good accordance with recently reported molecular dynamics simulations [5], which showed specific binding of Leg2 through interactions between the acyl chains of the bacterial membrane and the hydrophobic side chains of the peptide. Zhao et al. obtained similar results in molecular dynamics simulations predicting stronger interaction of the antimicrobial peptides LL-37 with bacterial membranes than with mammalian membranes [54]. In comparison to the peptide nisin, which is approved for food preservation, Leg1 and Leg2 ensured higher cell viability. Cytotoxic effects, if any, of Leg1/Leg2 were only observed at high concentrations. Therefore, the AMPs are nontoxic to a human cell line, which is an important prerequisite for the application of Leg1 and Leg2 as food preservatives.

## 4. Conclusions

In summary, the present study revealed additional beneficial properties of the known antimicrobial peptides Leg1 and Leg2 for a potential application as food preservatives. Thus, strong additive effects in combination with the preservative sodium benzoate may enable to reduce effective doses. Additionally, both AMPs inhibited the growth of two food-spoilage yeast strains. Moreover, especially Leg2 showed antioxidative effects by radical scavenging and reducing activity. Thus, the application of the plurifunctional peptides Leg1 and Leg2 may simultaneously hinder bacterial, fungal and oxidative mechanisms of food spoilage. Further studies are now required to test the activity and stability of Leg1 and Leg2 during food storage.

## Figures and Tables

**Table 1:** Results of the checkerboard assay of Leg1/Leg2/nisin and sodium benzoate (SB) against *E. coli* displaying MIC_A_/MIC_B_ (MIC for the peptide or SB), MIC_AB_/MIC_BA_ (MIC for the combination of peptide and SB) and fractional inhibitory concentration (FIC) values.

Peptide	MIC_A_/MIC_AB_ (Peptide) [µM]	MIC_B_/MIC_BA_ (SB) [µM]	FIC ^1^
Leg1	62.5/31.3	80,000/10,000	0.625
Leg2	62.5/31.3	80,000/10,000	0.625
Nisin	125/15.6	80,000/40,000	0.625

^1^ FIC < 0.5 interpreted as synergy, 0.5 < FIC > 2.0 as additivity, 2.0 < FIC > 4.0 as indifference and FIC > 4.0 as antagonism.

**Table 2:** Results of the checkerboard assay of Leg1/Leg2/nisin and sodium benzoate (SB) against *B. subtilis* displaying MIC_A_/MIC_B_ (MIC for the peptide or SB), MIC_AB_/MIC_BA_ (MIC for the combination of peptide and SB) and fractional inhibitory concentration (FIC) values.

Peptide	MIC_A_/MIC_AB_ (Peptide) [µM]	MIC_B_/MIC_BA_ (SB) [µM]	FIC ^1^
Leg1	15.6/7.8	80,000/10,000	0.75
Leg2	15.6/7.8	80,000/10,000	0.75
Nisin	7.8/2.0	80,000/40,000	0.75

^1^ FIC < 0.5 interpreted as synergy, 0.5 < FIC > 2.0 as additivity, 2.0 < FIC > 4.0 as indifference and FIC > 4.0 as antagonism.

**Table 3:** Results of the antioxidant DPPH assay using Leg1/Leg2 and single amino acids in a concentration range of 15.6–1000 µM. Ascorbic acid was used as positive control. The mean ± SD of triplicates of the antioxidative activity is displayed.

Concentration [µM]	Antioxidant Activity (%)						
	1000	500	250	125	62.5	31.3	15.6
Leg1 (RIKTVTSFDLPALRFLKL)	29.3 ± 3.7	6.0 ± 8.2	0.30 ± 0.5	- ^1^	-	-	-
Leg2 (RIKTVTSFDLPALRWLKL)	91.5 ± 8.3	72.2 ± 12.8	46.2 ± 16.6	20.9 ± 22.6	26.7 ± 1.3	17.7 ± 2.3	5.3 ± 3.3
Tyrosine	50.3 ± 6.0	52.4 ± 4.5	42.3 ± 11.6	31.2 ± 4.5	40.1 ± 7.5	10.4 ± 12.2	-
Histidine	34.5 ± 9.1	32.8 ± 7.3	30.0 ± 3.2	17.9 ± 11.9	19.1 ± 3.7	6.1 ± 4.0	0.2 ± 6.4
Cysteine	93.6 ± 1.9	82.6 ± 8.9	95.7 ± 1.5	94.1 ± 2.2	90.2 ± 8.8	88.5 ± 7.2	72.8 ± 11.6
Tryptophan	60.8 ± 11.1	38.6 ± 2.5	22.8 ± 4.4	15.2 ± 7.9	17.9 ± 5.8	19.5 ± 5.6	17.6 ± 5.7
Proline	28.7 ± 9.4	25.6 ± 10.9	23.3 ± 5.9	19.0 ± 11.6	8.5 ± 8.5	-	-
Phenylalanine	29.0 ± 6.2	20.5 ± 3.9	18.1 ± 8.4	19.3 ± 14.2	10.6 ± 6.4	-	-
Ascorbic acid	91.2 ± 2.4	82.2 ± 8.2	74.3 ± 13.2	79.2 ± 15.4	70.1 ± 4.2	71.9 ± 6.9	58.9 ± 1.9

^1^ (-) no antioxidative activity detected.

**Table 4:** Results of the reducing power assay using Leg1/Leg2 and single amino acids in a concentration range of 15.6–1000 µM. Ascorbic acid was used as the positive control. The mean ± SD of triplicates of the antioxidative activity is displayed.

Concentration [µM]	Antioxidant Activity (%)						
	1000	500	250	125	62.5	31.3	15.6
Leg1 (RIKTVTSFDLPALRFLKL)	70.3 ± 2.2	64.7 ± 3.0	56.3 ± 2.2	42.6 ± 3.0	26.0 ± 2.5	19.3 ± 1.4	8.3 ± 1.8
Leg2 (RIKTVTSFDLPALRWLKL)	86.3 ± 2.2	84.2 ± 3.3	79.5 ± 0.3	60.2 ± 2.4	52.4 ± 2.8	30.0 ± 1.7	20.3 ± 10.2
Tyrosine	87.1 ± 0.7	82.7 ± 0.7	75.1 ± 0.8	57.1 ± 1.9	40.9 ± 8.0	14.6 ± 2.8	11.1± 1.0
Histidine	85.4 ± 5.7	81.1 ± 0.9	30.0 ± 6.3	17.3 ± 3.2	19.0 ± 7.0	6.1 ± 2.6	- ^1^
Cysteine	90.2 ± 0.1	83.5 ± 0.1	75.4 ± 0.3	62.6 ± 0.4	47.5 ± 0.5	32.0 ± 1.8	20.0 ± 1.4
Tryptophan	92.7 ± 0.3	88.2 ± 1.0	82.2 ± 1.9	69.9 ± 5.4	55.7 ± 6.9	24.7 ± 6.2	10.8 ± 10.7
Proline	12.2 ± 4.2	28.2 ± 5.2	1.7 ± 4.8	33.6 ± 4.9	14.3 ± 1.3	-	-
Phenylalanine	22.8 ± 2.9	19.7 ± 0.5	14.1± 1.1	2.7 ± 3.1	-	-	-
Ascorbic acid	94.7 ± 0.1	92.0 ± 0.3	83.6 ± 0.6	74.6 ± 0.4	60.2 ± 1.7	40.8 ± 2.1	28.4 ± 0.9

^1^ (-) no antioxidative activity detected.

## Data Availability

Data available on request.

## Supplementary Materials

The following are available online at https://www.mdpi.com/2304-8158/10/3/585/s1, Table S1: Antifungal susceptibility tests of Leg1, Leg2 and nisin against *A. niger* using a microdilution assay; Table S2: Antifungal susceptibility tests of Leg1, Leg2 and nisin against *P. membranifaciens* using a microdilution assay; Figure S1: OD_620_ values of the blank controls consisting of water, YM and different concentrations of Leg1 or Leg2 (1–1000 µM).
