# Supplementary material for: Antioxidative, Antifungal and Additive Activity of the Antimicrobial Peptides Leg1 and Leg2 from Chickpea
Source: Foods. 2021 Mar 11;10(3):585. doi: 10.3390/foods10030585 (PMC7998185; doi:10.3390/foods10030585)
Supplement: Supplementary file 1 [file foods-10-00585-s001.pdf]

Supplementary Material

Antioxidative, Antifungal and Additive Activity of the Antimicrobial Peptides Leg1 and Leg2 from Chickpea

Marie-Louise Heymich [1], Laura Nißl [2], Dominik Hahn [1], Matthias Noll [2] and Monika Pischetsrieder [1,*]

[1] Food Chemistry, Department of Chemistry and Pharmacy, Friedrich-Alexander Universität Erlangen-Nürnberg (FAU), Nikolaus-Fiebiger-Str. 10, 91058 Erlangen, Germany; marie-louise.heymich@fau.de; dominik.hahn@fau.de; monika.pischetsrieder@fau.de
[2] Institute for Bioanalysis, Department of Applied Sciences, Coburg University of Applied Sciences and Arts, Friedrich-Streib-Str. 2, 96450 Coburg, Germany; lauranissl@web.de; matthias.noll@hs-coburg.de
* Correspondence: monika.pischetsrieder@fau.de;

Table S1. Antifungal susceptibility tests of Leg1, Leg2 and nisin against *A. niger* using a microdilution assay. Fungal growth was monitored at ΔOD_{620nm} and compared to negative control (OD_{620nm} = 2.4; *n* = 3). For comparison, sodium benzoate was tested accordingly (*n* = 3).

	**OD_{620nm} values**										
**Concentration [µM]**	**1000**	**500**	**250**	**125**	**62.5**	**31.3**	**15.6**	**7.8**	**3.9**	**2.0**	**1.0**
Leg1	2.4	2.3	2.2	2.3	2.3	2.2	2.3	2.2	2.3	2.2	2.1
Leg2	2.3	2.4	2.3	2.0	2.2	2.3	2.2	2.2	2.1	2.2	2.2
Nisin	3.5	3.4	3.5	3.1	2.9	2.4	2.4	2.9	2.4	2.6	2.5
**Concentration [µM]**	**160,000**	**80,000**	**40,000**	**20,000**	**10,000**	**5000**	**2500**	**1250**	**625**	**312.5**	**156.3**
Sodium benzoate	0.1	0.1	0.1	0.1	1.8	3.1	2.9	2.3	2.2	2.5	2.5

Table S2. Antifungal susceptibility tests of Leg1, Leg2 and nisin against *P. membranifaciens* using a microdilution assay. Fungal growth was monitored at ΔOD_{620nm} and compared to negative control (OD_{620nm} = 2.1; *n* = 3). For comparison, sodium benzoate was tested accordingly (*n* = 3).

	OD_{620nm} values										
**Concentration [µM]**	**1000**	**500**	**250**	**125**	**62.5**	**31.3**	**15.6**	**7.8**	**3.9**	**2.0**	**1.0**
Leg1	1.5	1.5	1.2	2.0	1.7	1.8	1.5	2.1	2.2	2.0	2.1
Leg2	1.4	1.8	1.7	1.9	2.1	2.2	1.7	1.5	1.9	1.6	1.9
Nisin	1.6	1.5	1.3	1.8	2.1	2.1	2.0	1.9	2.2	2.2	2.1
**Concentration [µM]**	**160,000**	**80,000**	**40,000**	**20,000**	**10,000**	**5000**	**2500**	**1250**	**625**	**312.5**	**156.3**
Sodium benzoate	0.1	0.3	0.4	0.8	1.1	1.8	2.0	2.0	2.1	2.1	2.1

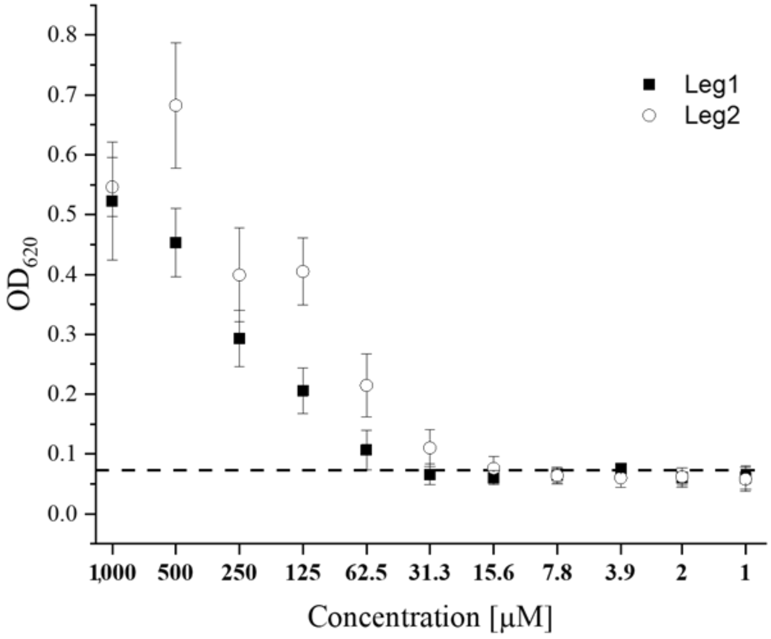

Figure S1. Optical density $(OD)_{620}$ values of the blank controls consisting of water, yeast/molds (YM) agar and different concentrations of Leg1 or Leg2 (1–1 000 μM). The means ± SD of triplicates are displayed. Water instead of the antimicrobial peptides was used as negative control (dashed line). The figure shows significant differences compared to the negative control.
